# Exploring Heavy Metal and Metalloid Exposure in Children: A Pilot Biomonitoring Study near a Sugarcane Mill

**DOI:** 10.3390/toxics12060426

**Published:** 2024-06-12

**Authors:** Oliver Mendoza-Cano, Agustin Lugo-Radillo, Mónica Ríos-Silva, Irma Elizabeth Gonzalez-Curiel, Jaime Alberto Bricio-Barrios, Arlette A. Camacho-delaCruz, María Fernanda Romo-García, Herguin Benjamín Cuevas-Arellano, Ana Luz Quintanilla-Montoya, Ramón Solano-Barajas, Juan Manuel Uribe-Ramos, Luis A. García-Solórzano, Ángel Gabriel Hilerio-López, Alma Alejandra Solano-Mendoza, Rogelio Danis-Romero, Efrén Murillo-Zamora

**Affiliations:** 1 Facultad de Ingeniería Civil, Universidad de Colima, Carretera Colima-Coquimatlán km 9, Col. Jardines del Llano, Coquimatlán 28400, Mexico; 2 CONAHCyT-Facultad de Medicina y Cirugía, Universidad Autónoma Benito Juárez de Oaxaca, Ex Hacienda Aguilera S/N, Carr. a San Felipe del Agua, Oaxaca 68020, Mexico; 3 Facultad de Medicina, Universidad de Colima, Av. Universidad 333, Col. Las Víboras, Colima 28040, Mexico; 4 Laboratorio de Inmunotoxicología, Unidad Académica de Ciencias Químicas, Universidad Autónoma de Zacatecas, Campus UAZ siglo XXI, Carretera Zacatecas-Guadalajara km 6, Col. Ejido La Escondida, Zacatecas 98160, Mexico; 5 Facultad de Ciencias, Universidad de Colima, Bernal Díaz del Castillo No. 340, Col. Villas San Sebastián, Colima 28045, Mexico; 6 Tecnológico Nacional de México, Campus Colima, Av. Tecnológico No. 1, Villa de Álvarez 28976, Mexico; 7 Facultad de Enfermería, Universidad de Colima, Av. Universidad 333, Colima 28040, Mexico; 8 Departamento de Medicina Interna, Hospital Civil de Guadalajara “Juan I. Menchaca”, Universidad de Guadalajara, Salvador Quevedo y Zubieta 750, Col. Independencia Oriente, Guadalajara 44340, Mexico; 9 Departamento de Pediatría, Hospital General Regional No. 1, Instituto Mexicano del Seguro Social, Av. 5 de Febrero 102, Col. Centro, Santiago de Querétaro 76000, Mexico; 10 Unidad de Investigación en Epidemiología Clínica, Instituto Mexicano del Seguro Social, Av. Lapislázuli 250, Col. El Haya, Villa de Álvarez 28984, Mexico

**Keywords:** child, metals, heavy, metalloids, environmental exposure

## Abstract

Sugarcane production has been linked to the release of heavy metals and metalloids (HM/MTs) into the environment, raising concerns about potential health risks. This study aimed to assess the levels of 19 HM/MTs in children living near a sugarcane mill through a pilot biomonitoring investigation. We investigated sex-related differences in these element levels and their correlations. A cross-sectional study was conducted, analyzing data from 20 children in the latter part of 2023. Spearman correlation coefficients with 95% confidence intervals (CIs) were used to assess the relationships between urinary HM/MT levels. Detectable levels of 17 out of the 19 HM/MTs were found across the entire study sample, with arsenic and copper detectable in 95% of the children. Titanium exhibited higher levels in boys compared to girls (p = 0.017). We identified 56 statistically significant correlations, with 51 of them being positive, while the remaining coefficients indicated negative relationships. This study characterized HM/MT levels in school-aged children residing near a sugarcane mill through a pilot biomonitoring investigation. Further research employing larger sample sizes and longitudinal assessments would enhance our understanding of the dynamics and health impacts of HM/MT exposure in this vulnerable population.

## 1. Introduction

Sugarcane production is a significant economic activity in many regions worldwide, including Mexico, where it is concentrated in coastal plains [1]. However, this industry can also pose environmental challenges. Various stages of sugarcane cultivation, processing, and waste disposal have the potential to release heavy metals and metalloids (HM/MTs) into the environment [2]. Mechanisms facilitating the release of these substances include the application of metal-containing fertilizers or pesticides during sugarcane cultivation and the inadequate disposal of solid waste generated from both cultivation and milling processes [3].

These HM/MTs pose potential health risks to nearby populations, particularly children [4]. They are more susceptible due to their developing bodies and higher intake rates relative to their body weight [5].

High levels of cadmium, copper, lead, and zinc have been documented in particulate matter from sugarcane mills [6]. However, most of these studies have focused on analyzing occupational exposure to these substances [7,8,9,10]. To the best of our knowledge, no published studies have examined this potentially hazardous exposure in populations without occupational contact with HM/MTs.

School-aged children residing near sugarcane mills are a population of particular concern due to their potential for increased exposure to HM/MTs through various pathways, including the inhalation of contaminated dust, ingestion of contaminated water or food, and dermal contact with contaminated soil or surfaces [11]. This potential exposure raises concerns about potential health consequences. These xenobiotics have been linked to various adverse health outcomes, including neurodevelopmental impairments, cognitive dysfunction, and chronic diseases [12].

This study aimed to assess the concentrations of HM/MTs in children living near a sugarcane mill, through a preliminary biomonitoring investigation, in the suburban locality of Quesería (population approximately 9900), located in the northern part of the state of Colima, Mexico. Additionally, we evaluated sex-related differences in the levels of the evaluated elements, as well as their correlations.

The findings of this study may provide valuable insights into the potential exposure of school-aged children to HM/MTs near sugarcane mills and contribute to the understanding of potential health risks associated with such exposure. This information can inform public health initiatives aimed at mitigating exposure and protecting children’s health in communities with similar characteristics.

## 2. Materials and Methods

An observational, cross-sectional epidemiological study was conducted in the second half of 2023. School-aged children (5–12 years old) residing within the participating locality for at least 5 years, as reported by their parents or legal guardians, were eligible to participate. Children with chronic non-communicable diseases and those who did not provide informed consent/assent were excluded.

**Figure 1:**
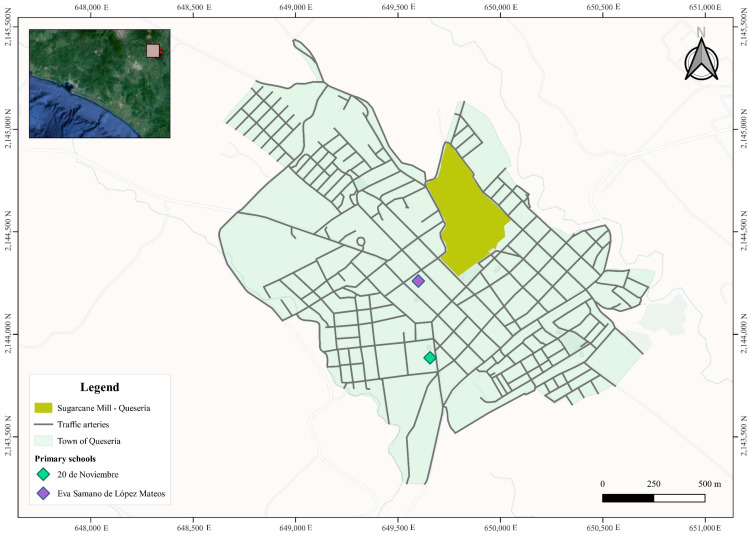
Location of the sugarcane mill and participating elementary schools in Quesería, Mexico 2023.

A non-probability sampling method was used to recruit participants. Children attending any of two local elementary schools (Figure 1) were invited to participate in an informative workshop through a written invitation. During the workshop, researchers provided detailed explanations of this study and obtained informed consent from parents or guardians and assent from the children themselves.

Anthropometric measurements, including weight and height, were taken in this study. Z scores for body mass index (BMI) for age were calculated to assess the participants’ nutritional status. These Z scores were computed using the WHO Child Growth Standards.

A structured questionnaire was utilized to gather data on the individuals’ personal medical history. The instrument comprised closed-ended questions and was administered by trained personnel in a private classroom. This approach ensured that interviewers performed tasks or measurements consistently and uniformly, reducing variability in the data collection process. The interview was conducted with the mother in 60% of cases.

After obtaining informed consent and child assent, participants provided a morning urine sample through spontaneous micturition. The urine was collected in sterile 150 mL containers and stored at −70 °C until analysis. Subsequently, the urine samples were thawed, and aliquots of 200 μL were directly diluted 1:10 with 65% nitric acid (Merck, Darmstadt, Hesse, Germany) and ultrapure water to achieve a final concentration of 0.16%. The samples were then analyzed using an ICP-MS NexION 300D (Perkin Elmer, Waltham, MA, USA). The equipment was optimized for each run according to the manufacturer’s instructions.

The quantification was performed using a validated method (LISTO-MET-PRO-TEC-016) from the Metal Laboratory of the Research and Service Laboratory in Toxicology of the Center for Research and Advanced Studies of the National Polytechnic Institute (CINVESTAV). The determinations were conducted in duplicate, including a calibration curve with a blank and at least six different concentrations (0.5, 1, 5, 10, 25, 50, and 100 ng/mL) from the multi-element calibration standards 2, 3, 4, and 5 (Perkin Elmer, Waltham, MA, USA).

As an analytical quality control measure, the precision and accuracy of the determinations were evaluated, ensuring that the analytical coefficient of variation was not greater than 10% in the duplicates of the sample. For the evaluation of accuracy, certified reference materials for urine were utilized—QM-U-Q2104, 105, 106, 113, 114, and 115—from the Institut National de Santé Publique du Québec (INSPQ). These materials were analyzed together with the study samples, yielding a percentage of accuracy in the analysis between 80% and 120%. The results were adjusted for urine specific gravity, and any undetected samples were assigned the value of the limit of detection (LOD), as per the Guidance for Data Quality Assessment [13].

Summary statistics were computed, categorized by sex. A significance level of 5% was used to assess the statistical differences between groups. We calculated Spearman’s rank correlation coefficients (ρ) along with 95% confidence intervals (CIs) to assess potential relationships between the different HM/MTs measured.

To maintain the confidentiality of the schools where the children were recruited, a cluster analysis was performed and no results were shown for each school. The study protocol was reviewed and approved by the Committee of Ethics in Health Research of the Ministry of Health in the state where the research was conducted (approval number: CBCANCL2306023-PRONAII-17).

## 3. Results

This study included 20 participants (8 girls and 12 boys) (Table 1), with 8 children enrolled from one school and 12 from the other. No statistically significant differences in sex distribution were observed between the schools in the study sample (p = 0.142).

Their median age was 9 years old (interquartile range [IQR] 7–11 years), and no significant differences in age were observed between girls and boys (p = 0.635). Half (50%) of the participants had a normal BMI for their age, and its prevalence was similar between girls and boys (p = 0.212).

Among the participants, 15% (3 out of 20) reported a personal history of chronic respiratory diseases. Additionally, 20% (4 out of 20) reported possessing a neurologic disorder or a psychiatric disorder. The prevalence of allergic disorders was 10% (2 out of 20). No participants reported a history of endocrine or renal diseases.

Detectable levels of 17 out of the 19 analyzed HM/MTs were observed in the entire study sample. The exceptions were arsenic (LOD ≤ 0.041 ng/mL) and copper (LOD ≤ 0.112 ng/mL), which were detectable in 95% of the children.

The total and sex-stratified levels (ng/mL) of the HM/MTs are summarized in Table 2. No significant differences were documented for most of the elements, except for titanium. Median titanium levels were higher in boys (32.3 ng/mL, 18.3–46.9) than in girls (15.4 ng/mL, 10.5–22.8), with a statistically significant difference (p = 0.017).

In the Spearman correlation analysis, we identified a total of 56 statistically significant coefficients (Table 3). Most of these (51 out of 56) indicated positive correlations, while the remaining coefficients represented negative associations. The strongest positive correlations were observed between cesium and lithium (ρ = 0.91, 95% CI 0.77 to 0.99; p < 0.001), molybdenum and cadmium (ρ = 0.88, 95% CI 0.70 to 0.99; p < 0.001), iron and strontium (ρ = 0.85, 95% CI 0.67 to 0.99; p < 0.001), and cesium and arsenic (ρ = 0.85, 95% CI 0.66 to 0.99; p < 0.001).

Among the 5 negative and significant correlations, 3 involved the relationship of nickel with selenium (ρ = −0.52, 95% CI −0.87 to −0.16; p = 0.004), titanium (ρ = −0.69, 95% CI −0.92 to −0.47; *p* < 0.001), and zinc (ρ = −0.56, 95% CI −0.89 to −0.22, p = 0.001).

Cesium exhibited the highest number of significant correlation coefficients, with 13, followed by arsenic, with 12.

## 4. Discussion

Our study characterized HM/MT levels in school-aged children residing near a sugarcane mill through a pilot biomonitoring investigation. Our results provide evidence of the exposure of the analyzed children to the assessed substances; however, the potential limitations of the study design must be considered in the interpretation of our findings.

Arsenic and lead were detected in 95% and 100% of the enrolled children, respectively. These findings align with previous documentation of arsenic and lead exposure in Chinese children residing in rural areas with sugarcane-related activities [14]. To the best of our knowledge, this latter study is the only published research focusing specifically on children as we have done. The presented results also align with the levels of HM/MTs registered in soils around a sugarcane mill in the Mexican state of Veracruz (cadmium, copper, lead, and zinc), which range from 50 to 900 m from the mill [6].

We found similar concentrations of most heavy metals and metalloids (HM/MTs) in the urine of boys and girls. However, an exception was titanium, with boys exhibiting significantly higher levels compared to girls. This sex disparity in urinary titanium warrants further investigation to identify potential sources and health implications, especially considering the established toxicological effects of titanium compounds [15].

In the environment, the titanium can arise from natural sources, such as soil and rocks, as well as anthropogenic activities, including industrial emissions and waste disposal [16]. Therefore, the elevated levels of titanium observed in boys could be attributed to differential exposure patterns related to lifestyle, occupational activities, or dietary habits [17]. However, animal studies have documented sex differences in the liver toxicity of titanium nanoparticles [18].

The correlation analysis revealed numerous statistically significant coefficients, predominantly indicating positive correlations among various HM/MTs. However, these findings must be considered with caution due to the limited sample size.

Of particular note were the strong positive correlations observed between cesium and lithium, molybdenum and cadmium, and strontium and iron. These associations may reflect shared sources of exposure or biological interactions among these elements within the local environment [19]. Furthermore, published data support this notion, as studies have documented that crops irrigated with wastewater from sugar mills can accumulate cadmium, zinc, manganese, iron, copper, and nickel at levels exceeding health risk standards, particularly in vegetables like cilantro or coriander (*Coriandrum sativum*) [20].

Cesium emerged with the highest number of significant correlation coefficients, underscoring its importance in the context of environmental exposure assessment and potential health implications. Similarly, arsenic also demonstrated substantial associations, indicating their relevance to the local environmental and health considerations.

The simultaneous exposure to various metals has the ability to alter the risk of suffering from different diseases. In this sense, identifying the correlations between the metals detected in the same individual or population could be very useful for focusing efforts on preventing diseases associated with specific combinations of metals, previously documented in the scientific literature [21,22,23].

In this study, we employed specific gravity adjustment to account for variations in urine concentration, which can affect the measured levels of certain substances, particularly inorganic compounds such as heavy metals [24]. This adjustment helped standardize the results, making them more comparable across different urine samples. Creatinine adjustment is more commonly used for organic compounds, such as pesticides, where the goal is to account for variations in urine dilution and correct for differences in excretion rates due to differences in muscle mass [25]. However, creatinine adjustment may not be necessary or relevant for HM/MTs, as their excretion rates are less influenced by muscle mass and more related to other factors such as exposure levels [26].

Published data regarding the HM/MT levels in children are numerous, as are the number of detected substances [27,28,29,30,31], and most of the sources have been identified as anthropogenic [32]. The majority of published studies in this field have been conducted in Asia [33]. To the best of our knowledge, no published data have examined these levels in children in close contact to the emissions of a sugarcane mill. Moreover, the exposure to a mixture of substances has been associated with particular health outcomes. For example, in a Chinese population of children, the simultaneous exposure to lead, cadmium, mercury, and arsenic was associated with an impaired immune response and inflammatory regulation [34].

This study benefited from several strengths. We investigated a broad range of HM/MTs, performed sex-specific analyses, and explored potential relationships between different elements through correlation analysis. However, some limitations are important to acknowledge. The analysis of HM/MTs is expensive, and with a sample size of only 20 participants, the findings may not be generalizable to a wider population. Additionally, the non-probabilistic selection of participants introduces the possibility of selection bias. Furthermore, while the correlations between HM/MT levels are informative, they cannot establish a cause-and-effect relationship with health outcomes.

Second, the small sample size limits the viability of conducting a more robust analysis, such as principal components analysis, which might provide a more comprehensive view of the correlation structure among urinary levels of multiple HM/MTs, aiding in the identification of common exposure patterns. Finally, in this first exploratory biomonitoring study, we did not analyze potential exposure routes (i.e., specific food consumption), which would have provided a deeper characterization of the HM/MT exposure in the analyzed children.

## 5. Conclusions

Our study provides insights into the HM/MT exposure profile of school-aged children in the vicinity of a sugarcane mill in Quesería, Colima, Mexico. The observed correlations and sex-related differences underscore the need for continued monitoring and targeted interventions to mitigate potential health risks associated with environmental exposures to these elements. Further research incorporating larger sample sizes and longitudinal assessments would enhance our understanding of the dynamics and health impacts of HM/MT exposure in this vulnerable population.

## Figures and Tables

**Table 1:** Characteristics of the study sample for selected variables, Mexico 2023.

Characteristic	Overall	Girls	Boys	p
	n = 20	n = 8	n = 12	
Age (median, IQR), years	9 (7–11)	9 (7–11)	9 (8–10)	0.635
Age group (tertiles), years				
5–7	6 (30.0)	3 (37.5)	3 (25.0)	0.535
8–9	8 (40.0)	2 (25.0)	6 (50.0)	
10–12	6 (30.0)	3 (37.5)	3 (25.0)	
Height (median, IQR), cm	136.2 (129.4–143.7)	139.0 (128.6–146.7)	134.0 (129.8–142.8)	0.700
Weight (median, IQR), kg	32.9 (24.5–48.4)	29.0 (23.6–45.6)	32.3 (27.5–48.4)	0.488
BMI-for-age				
Low	1 (5.0)	1 (12.5)	0 (0)	0.212
Normal	10 (50.0)	5 (62.5)	5 (41.7)	
Overweight–Obesity	9 (45.0)	2 (25.0)	7 (58.3)	

Abbreviations: IQR, interquartile range. Notes: (1) The absolute (n) and relative (%) frequencies are presented, unless the median and IQR are specified. (2) The p-values from chi-squared or U tests for comparisons between girls and boys are presented accordingly. (3) The nutritional status was evaluated using Z scores determined with the WHO Child Growth Standards.

**Table 2:** Urinary levels of analyzed heavy metals and metalloids in school-aged children, Mexico 2023.

	Overall	Girls	Boys	p
Aluminum	7.9 (4.7–13.3)	7.0 (5.0–13.5)	8.4 (4.7–13.2)	0.877
Arsenic	13.4 (5.9–19.6)	8.4 (1.0–19.3)	15.0 (7.4–19.6)	0.280
Barium	1.8 (1.0–2.5)	1.8 (0.8–2.8)	1.8 (1.1–2.4)	0.999
Cadmium	0.33 (0.28–0.37)	0.34 (0.27–0.35)	0.31 (0.28–0.42)	0.615
Cesium	8.0 (4.6–11.0)	7.9 (3.3–8.5)	8.8 (5.7–12.3)	0.165
Cobalt	1.2 (0.8–1.6)	0.9 (0.7–1.3)	1.3 (1.1–1.7)	0.177
Copper	9.6 (4.3–19.0)	12.0 (2.9–17.5)	8.5 (4.3–23.5)	0.758
Iron	401.5 (218.4–480.5)	311.4 (170.9–465.0)	403.6 (277.9–520.1)	0.316
Lead	1.8 (1.5–2.0)	1.6 (1.2–2.0)	1.8 (1.6–2.0)	0.316
Lithium	41.9 (30.6–53.6)	39.8 (23.5–43.5)	44.4 (32.8–61.1)	0.123
Mercury	0.5 (0.4–0.6)	0.5 (0.4–0.7)	0.5 (0.4–0.6)	0.699
Molybdenum	93.6 (63.4–131.2)	86.2 (60.3–100.6)	107.7 (70.1–178.1)	0.123
Nickel	7.6 (4.8–9.1)	8.4 (6.5–9.1)	5.8 (4.4–9.3)	0.440
Selenium	63.6 (56.7–87.4)	67.7 (55.4–69.5)	60.3 (56.7–109.8)	0.817
Strontium	171.2 (128.3–261.9)	158.0 (74.7–267.6)	180.7 (131.5–261.9)	0.537
Tellurium	0.59 (0.58–0.61)	0.61 (0.58–0.62)	0.59 (0.58–0.61)	0.368
Tin	1.3 (0.5–2.0)	1.2 (0.3–2.0)	1.3 (0.5–2.0)	0.758
Titanium	22.0 (15.4–35.7)	15.4 (10.5–22.8)	32.3 (18.3–46.9)	0.017
Zinc	604.8 (427.1–822.0)	597.3 (506.3–822.0)	617.9 (415.2–820.7)	0.939

Abbreviations: IQR, interquartile range. Note: the p-values from U tests for comparisons between girls and boys are presented.

**Table 3:** Relationship between the urinary levels of heavy metals and metalloids in school-aged children, Mexico 2023.

	Al	As	Ba	Cd	Cs	Co	Cu	Fe	Pb	Li	Hg	Mo	Ni	Se	Sr	Sn	Ti	Te	Zn
Al	1.00																		
As	0.76 *	1.00																	
Ba	0.34	0.63 *	1.00																
Cd	0.36	0.70 *	0.40	1.00															
Cs	0.53 *	0.85 *	0.66 *	0.56 *	1.00														
Co	0.04	0.33	0.56 *	0.27	0.48 *	1.00													
Cu	0.41	0.52 *	0.45 *	0.30	0.52 *	0.39	1.00												
Fe	0.36	0.43	0.42	0.27	0.43	0.22	0.24	1.00											
Pb	0.34	0.50 *	0.37	0.61 *	0.29	0.23	0.31	0.45 *	1.00										
Li	0.38	0.79 *	0.65 *	0.65 *	0.91 *	0.50 *	0.39	0.39	0.34	1.00									
Hg	0.05	0.14	0.13	−0.01	0.16	0.07	0.43	0.10	−0.01	0.07	1.00								
Mo	0.13	0.54 *	0.24	0.88 *	0.54 *	0.32	0.27	0.22	0.57*	0.58 *	−0.01	1.00							
Ni	−0.07	−0.02	0.28	−0.09	−0.02	−0.07	−0.14	−0.09	0.17	−0.17	−0.29	0.02	1.00						
Se	0.55 *	0.64 *	0.35	0.48 *	0.57 *	0.19	0.66 *	0.23	0.12	0.59 *	0.50 *	0.22	−0.52 *	1.00					
Sr	0.55 *	0.64 *	0.73 *	0.41	0.57 *	0.41	0.41	0.85 *	0.54 *	0.48 *	0.11	0.28	0.12	0.32	1.00				
Sn	0.79 *	0.65 *	0.40	0.34	0.62 *	0.01	0.22	0.58 *	0.39	0.48 *	−0.05	0.21	0.02	0.31	0.62 *	1.00			
Ti	0.09	0.18	−0.15	0.16	0.17	0.17	0.16	0.29	−0.08	0.34	0.20	0.15	−0.69 *	0.38	0.03	0.01	1.00		
Te	−0.17	−0.40	−0.18	−0.39	−0.52 *	−0.15	−0.08	−0.18	−0.29	−0.65 *	0.13	−0.34	0.32	−0.31	−0.07	−0.39	−0.24	1.00	
Zn	0.65 *	0.62 *	0.19	0.30	0.51 *	0.14	0.52 *	0.41	0.01	0.41	0.50 *	0.10	−0.56 *	0.82 *	0.40	0.41	0.43	−0.16	1.00

Abbreviations: Al (aluminum); As (arsenic); Ba (barium); Cd (cadmium); Cs (cesium); Co (cobalt); Cu (copper); Fe (iron); Pb (lead); Li (lithium); Hg (mercury); Mo (molybdenum); Ni (nickel); Se (selenium); Sr (strontium); Sn (tin); Ti (titanium); Te (tellurium); and Zn (zinc). Notes: (1) The Spearman’s regression coefficients (rho) are presented. (2) * p < 0.05.

## Data Availability

Requests for data sharing should be directed to the corresponding author and will undergo review by lead investigators and the funding council. Upon request, deidentified data and a data dictionary defining each field in the dataset will be made available after a proposal is approved and a data use agreement is signed.
